# Computerized Segmentation and Characterization of Breast Lesions in Dynamic Contrast-Enhanced MR Images Using Fuzzy c-Means Clustering and Snake Algorithm

**DOI:** 10.1155/2012/634907

**Published:** 2012-08-21

**Authors:** Yachun Pang, Li Li, Wenyong Hu, Yanxia Peng, Lizhi Liu, Yuanzhi Shao

**Affiliations:** ^1^School of Physics and Engineering, Sun Yat-sen University, Guangzhou 510275, China; ^2^Imaging Diagnosis and Interventional Center, Cancer Center, Sun Yat-sen University, Guangzhou 510060, China

## Abstract

This paper presents a novel two-step approach that incorporates fuzzy c-means (FCMs) clustering and gradient vector flow (GVF) snake algorithm for lesions contour segmentation on breast magnetic resonance imaging (BMRI). Manual delineation of the lesions by expert MR radiologists was taken as a reference standard in evaluating the computerized segmentation approach. The proposed algorithm was also compared with the FCMs clustering based method. With a database of 60 mass-like lesions (22 benign and 38 malignant cases), the proposed method demonstrated sufficiently good segmentation performance. The morphological and texture features were extracted and used to classify the benign and malignant lesions based on the proposed computerized segmentation contour and radiologists' delineation, respectively. Features extracted by the computerized characterization method were employed to differentiate the lesions with an area under the receiver-operating characteristic curve (AUC) of 0.968, in comparison with an AUC of 0.914 based on the features extracted from radiologists' delineation. The proposed method in current study can assist radiologists to delineate and characterize BMRI lesion, such as quantifying morphological and texture features and improving the objectivity and efficiency of BMRI interpretation with a certain clinical value.

## 1. Introduction

Breast cancer is the most common cancer and a leading cause of deaths in cancer for women worldwide [1]. In the United States, the chance of developing invasive breast cancer in a woman's life is nearly 1 in 8 [2]. Medical imaging, specifically, magnetic resonance imaging (MRI) plays a crucial role in detecting and diagnosing breast lesions and tumors. While mammography, as recently reported, might fail to spot up to 20 percent of tumors, MRI can detect breast cancer missed by mammography [3, 4]. Because of MRI's effectiveness in detecting breast cancer, American Cancer Society has published the guidelines for recommending women with high risk of breast cancer to receive MRI screening [5].

With its high sensitivity and variable specificity, MRI has been increasingly used for a breast cancer detection and characterization [6–8]. As a result, there is an urgent need to develop a computer-aided diagnosis system to release radiologists from the heavy works of medical image analysis. Unfortunately, compared with mammography, relatively fewer automated CADs have been developed specifically for breast MRI. Chen et al. [9] applied the region-growing method to segment lesions and later they [10] proposed a semiautomated algorithm based on the fuzzy c-means (FCMs) clustering with the shortcoming oversensitivity to noise; Liney et al. [11] presented a user-interaction-threshold method to extract the region of interest (ROI), requiring manual intervention; Stoutjesdijk et al. [12] designed an automated computer program to select the ROIs on the basis of a mean-shift-clustering method, the method is an accurate method to automatically determine a contiguous region of interest. Shi et al. [13] used the FCMs clustering algorithm followed by a 3D level set (LS) method for segmentation refinement, and a recent paper by Meinel et al. [14] reported a computerized segmentation method for mass-like breast MRI lesion involving robust seed-point selection, which is more reproducible than manual method in measuring the size and shape of a lesion. Because MR images are a sequence of two-dimensional images, the segmentation in 2D is still important and the basis of 3D segmentation.

Texture analysis is extensively utilized to quantify image characteristics (i.e., homogeneity and regularity with diagnosis potential in MR images). Gray level cooccurrence matrix (GLCM) method, proposed by Haralick et al. [15], is instrumental in medical image analysis. Various studies on texture analysis have been reported, including brain disease [16], bone [17], and abdominal tumor [18]. The GLCM method is also applied to the analysis of breast cancer. Chan et al. [19] put forward a method based on the texture features for discriminating mammography lesions by using linear discriminant analysis. Gibbs and Turnbull [20] manually delineated the breast MRI lesions, and then employed the GLCM method to differentiate benign and malignant lesions.

Computer-extracted morphological features have demonstrated to be of certain usefulness for characterizing breast lesions [11, 21, 22]. Breast Imaging Reporting and Data System (BI-RADS) lexicon has been introduced to categorize lesion appearance. However such lexicons are subject to radiologists' assessment. The objective computer-extracted features may benefit a radiologist to improve the interpretation and acceptability of a distinguishing feature.

In this study, we investigate systematically the segmentation and characterization of both benign and malignant breast lesions inside breast MR images using a computerized segmentation and characterization package we developed specifically for Breast MRI. The computational results of both segmentation and characterization of breast lesions are also compared with the manual delineation and the pathological results given by experienced radiologists.

## 2. Materials and Methods

**Figure 1:**
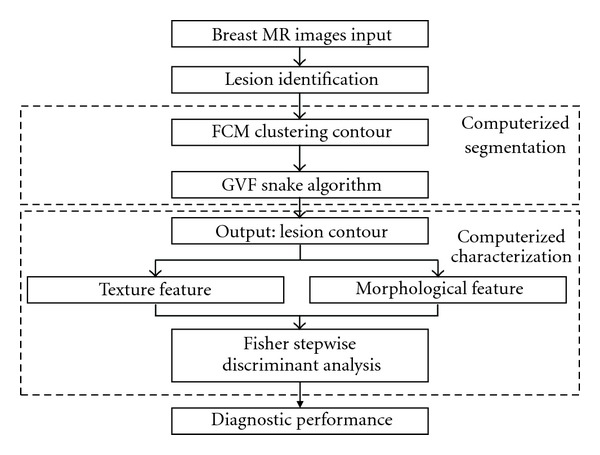
Flowchart of computerized lesion segmentation and characterization on breast MRI.

Figure 1 shows the flowchart for our computerized breast lesion segmentation and characterization method. Our computer program performs an automated segmentation and an image analysis consequently after the manual lesion identification of a breast MRI (2D) is input. In the computerized segmentation section, FCMs clustering based method is used to produce an initial segmentation of the input image, while the gradient vector flow (GVF) snake model is applied to the initial segmentation to obtain the final segmentation. The initial segmentation method is referred to as the FCMs-based and the final segmentation method is referred to as the GVF-FCMs for short. The segmentation performance of both methods is evaluated with manual segmentation by experienced radiologists on dynamic contrast-enhanced (DCE) MRI. In the computerized characterization section, we extract morphological and texture features from both the GVF-FCMs method and radiologists' delineation. Finally, Fisher stepwise discriminant analysis (FSDA) is applied to select the features extracted by the GVF-FCMs and the radiologists' manual delineation in differentiating between benign and malignant lesions.

### 2.1. Breast Lesions Database

This study consists of 22 benign and 38 malignant breast lesions which have been examined with a final histopathology confirmation (age range = 27–65 years old, mean age ± standard deviation = 42 ± 9 years). Only mass-like lesions that showed strong contrast enhancements were selected for this study. The database of the images for each case includes one sagittal postcontrast image slice that shows an obvious contrast enhancement and demonstrates the maximum dimension of a mass lesion. The size of the image is 512 × 512 pixels with a 256-gray level.

MR imaging was performed on a 1.5 T superconductive magnetic system (GE, Signa, HDx). A breast-specific 4-channel phased-array surface coil was used. Contrast medium was injected through a hand venipuncture technique. Patients were scanned in the prone position with bilateral breast naturally hanging into the two holes of the coil and their feet were first placed into the machine. A cross-sectional FSE T1WI was first employed using the following parameters: TR = 650 ms, TE: Min Full, ETL = 2, and BW = 20.83. The cross-sectional and sagittal FSE T2WI were then employed using the following parameters: TR = 4650 ms, TE = 85 ms, ETL = 16 and BW = 20.83, THK = 6 mm, spacing = 1 mm, and FOV was adjusted based on the breast size, ranging from 18 cm to 28 cm, matrix = 320 × 224, Frep DIR = A/P and NEX = 2. Except cross-sectional T1WI, all other sequences were fat suppression sequence. DCE-MRI was conducted after plain scan as following: (1) dynamic scanning was initiated after satisfied image quality was obtained in prescanning by simultaneously push the high-pressure syringe button and the dynamic scan button; (2) using MRI-specific high-pressure syringe (Medrad injector system, Pittsburgh) to inject 0.1 mmol/kg body weight contrast medium gadolinium diethylenetriamine penta-acetic acid (Gd-DTPA) using hand venipuncture technique at rate of 3 mL/s and then inject 10 mL saline at 3 mL/s to wash the tube; (3) all patients accepted sagittal vibrant multitemporal DCE-MRI using 3D Fast FSPGR pulse sequence and the following parameters: FA = 12, BW = 83.33, matrix = 288 × 288, FOV = 38 mm, phase FOV = 0.90, Frep DIR = A/P, multiphase = 8~10, Zip = 2, THK = 3.4 mm and locs per slab = 50 mm; (4) the initial section of the dynamic study was obtained in the sagittal plane at 20 second intervals for 11 minutes. After that, cross-sectional and sagittal MRI was employed using fat-suppressed enhanced T1WI sequence.

### 2.2. Initial Segmentation

**Figure 2:**
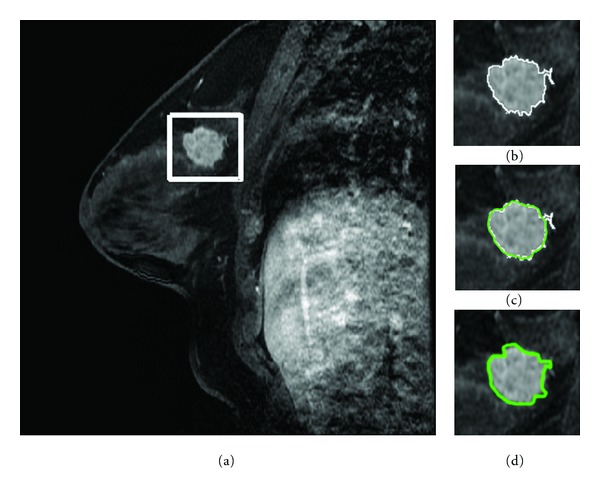
Lesion segmentation on a breast MRI scan: (a) locate a rectangle ROI box that contained a postcontrast breast MRI lesion; (b) initial segmentation by the FCMs-based method; (c) deformation of GVF snake using FCMs-based contour for initialization; (d) radiologists' manual delineation. The average time cost and dynamic memory cost of the method we proposed are 2.4180 seconds and 1256.75 KB.

Segmentation accuracy has a considerable influence on the subsequent characterization used to differentiate between benign and malignant breast lesions. Because of this reason, an experienced radiologist is included to identify the suspicious areas of breast lesions by firstly locating and defining a rectangle region of interest, as shown in Figure 2(a). The regions of interest serve as an input to the following sections. Then a two-step segmentation method is used to find out the accurate contour of a lesion. FCMs clustering based method is used to produce an initial segmentation of the ROI before the GVF snake for refinement is carried out.

The FCMs is an unsupervised machine learner in the pattern-recognition field and it has been widely used in image processing as well [23]. MR images always present overlapping intensities for different tissues because of the noise and blur in acquisition. The borders between different tissues are intrinsically fuzzy. The conventional (hard) clustering methods forces pixels to belong exclusively to one class. Therefore, fuzzy c-means clustering (FCMs) method allows uncertain belonging by a varying membership map and turns out to be particularly suitable for the segmentations of MR images.

In this study, the FCMs method is applied to the ROI for building the likelihood membership map (cluster number, 2; weighting exponent, 2; stop criteria, 0.0005, max iteration, 100). To binarize the membership map, we have referred to some articles [10, 13] and experimentally determined a likelihood threshold *T* = 0.5. Within the binary membership map, the processes including hole-filling, morphological opening, and two-dimensional connected-component labeling (8-connected objects) are carried out to remove the disconnections from the main lesion part. Finally, an initial segmentation is obtained with a slightly reduced size. Figure 2(b) shows an initial lesion segmentation using the FCMs-based method.

### 2.3. GVF Segmentation

In this study, the gradient vector flow (GVF) snake model is applied to further refine the initial segmentation. Here the word “snake” refers to a curve that can deform under the influence by both “internal” and “external” forces [24]. GVF snake model are commonly applied to medical images because they can capture the irregular shapes and shape deformations found in anatomical structures. Its main contributions are to overcome leakage at weak boundaries in progressing snakes into concave boundary regions. As for the GVF snake model, the external force field is defined as a diffusion of the gradient vectors of a gray-level edge map derived from the image [25]. The edge map *f*(*x*, *y*) derived from an image *I*(*x*, *y*) is defined as

(1) $$\begin{array}{c} f\left(x,y\right)={\left|\nabla I\left(x,y\right)\right|}^{2}. \end{array}$$

The GVF external field is the vector field **v**(*x*, *y*) = (*u*(*x*, *y*), *v*(*x*, *y*)) that minimizes the energy functional

(2) $$\begin{array}{c} \varepsilon =\iint \mu \left(u_{x}^{2}+u_{y}^{2}+v_{x}^{2}+v_{y}^{2}\right)+{\left|\nabla f\right|}^{2}{\left|v-\nabla f\right|}^{2}\;dxdy, \end{array}$$

where *μ* is a regularization parameter governing the tradeoff between the first term and the second term in (2). According to [25], we chose *μ* = 0.1 experimentally for the segmentation task in our study.

A GVF snake is a parameter curve defined as

(3) $$\begin{array}{c} x\left(s\right)=\left(x\left(s\right),y\left(s\right)\right), \end{array}$$

where *s* denotes an arc length parameter. The curve deforms iteratively until reaching a balance between the internal force **F**_int⁡_ and the external force **F**_ext_. The internal and external forces are

(4) $$\begin{array}{c} F_{\mathrm{int}}=\alpha x^{\prime \prime }\left(s\right)-\beta x^{\prime \prime \prime \prime }\left(s\right),\\ F_{\text{ext}}=v\left(x,y\right), \end{array}$$

where *α* and *β* are weighting parameters that control the snake's tension and rigidity and experimentally set as 0.01 and 0 according to [24, 25]. Double and quadruple primes represent the second- and fourth-order derivatives of **x**(*s*), respectively. The GVF snake model is solved numerically by discretization and iteration in similar fashion to the traditional snake [24]. In the iterative procedure, the internal force prevents the snake contour from stretching and bending excessive [25], while the external force pulls the snake toward the real contour. We will set the max iterations when the snake is iterating to reach a balance. It is hard to reach a balance when the image is quite blurred and complex. Figure 2(c) shows the deformation of the GVF snakes initialized by an FCMs-based method.

### 2.4. Feature Extraction

#### 2.4.1. Texture Features

Texture is one of the intrinsic characteristics of an object, and it is important for medical image analysis [26]. Various textural algorithms have been proposed by researchers, such as fractal-based description, texture spectrum, and Markov random field model [27–29]. The GLCM texture method is widely used in medical image processing through utilizing the relative positions of pixels [15]. The matrix element *p*_*θ*,*d*_(*i*, *j*) of the GLCM is the joint probability density of the occurrence for a pixel pair in an ROI with a defined distance *d*, direction *θ*, and gray levels *i* and *j*. We calculated thirteen textural measures for the nearest pixels (distance: 1 pixel) in four limited directions, 0°, 45°, 90° and 135°, respectively. Thirteen features derived from the GLCM are angular second moment, contrast, correlation, inverse difference moment, sum average, sum variance, sum entropy, entropy, difference average, difference variance, difference entropy, information measure of correlation 1, and information measure of correlation 2, respectively. Owing to the isotropic texture of the images investigated, the features we evaluated in the current study are the averages over the four directions. These texture features contain some important information on homogeneity, contrast, and other organized structures of images.

#### 2.4.2. Morphological Features

Eight morphological features, including compactness, spiculation, extent, elongation, solidity, circularity, and entropy of radial length distribution, are selected and computed to describe the morphological properties *f* as defined in the Breast Imaging Reporting and Data System lexicon. Listed below are the definitions of these features.

   
*p*_1_ : Compactness
  (5) $$\begin{array}{c} p_{1}=\frac{P^{2}}{4\pi S}, \end{array}$$
  where *P* and *S* are the perimeter length and area for a given breast MRI lesion contour, respectively.
   
*p*_2_: Spiculation
  (6) $$\begin{array}{c} p_{2}=\frac{1}{N}\sum_{i=1}^{N}\left|r_{i}-r_{i+1}\right|,\;\;r_{N+1}=r_{1}, \end{array}$$
  where *N* is the number of pixels on the lesion contour and *r*_*i*_ is the individual radial length. The individual radial length is defined as the Euclidean distance from the object's center to each of contour pixels.
   
*p*_3_: Extent
  (7) $$\begin{array}{c} p_{3}=\frac{S}{S_{\text{box}}}, \end{array}$$
  where *S*_box_ is the area of the smallest rectangle containing the given lesion contour.
   
*p*_4_: Elongation
  (8) $$\begin{array}{c} p_{4}=\frac{\min\left(H,L\right)}{\max\left(H,L\right)}, \end{array}$$
  where *H* and *L* are the vertical and horizontal length of the smallest rectangle containing the given lesion contour.
   
*p*_5_: Solidity
  (9) $$\begin{array}{c} p_{5}=\frac{S}{S_{\text{convex}}}, \end{array}$$
  where *S*_convex_ is the area of the smallest convex polygon that can contain the given lesion contour.
   
*p*_6_: Circularity
  (10) $$\begin{array}{c} p_{6}=\frac{1}{N}\sum_{i=1}^{N}r_{i}-\mu_{r}, \end{array}$$
  where *μ*_*r*_ is the average of *r*_*i*_.
  
*p*_7_: Entropy of radial length distribution
  (11) $$\begin{array}{c} p_{7}=-\sum p\left(r_{i}\right)\log\left(p\left(r_{i}\right)\right), \end{array}$$
  where *p*(*r*_*i*_) is the probability density of a given *r*_*i*_.
   
*p*_8_: Eccentricity
  Eccentricity is a scalar that specifies the eccentricity of the ellipse that has the same second-moments as the lesion region. It is the ratio of the distance between the foci of the ellipse and its major axis length.

### 2.5. Segmentation Performance Measure

It is somewhat difficult to appraise the segmentation performance of a computerized segmentation method, because there is no golden truth in delineating accurate contour. In this paper, we take the manual delineation by two experienced radiologists in interpreting BMRI as a reference standard. All images were manually delineated by the two radiologists who were blinded to the histological results, and the disagreements were resolved by consensus. Figure 2(d) demonstrates the delineation of the radiologists.

The lesion areas extracted by the FCMs-based initial segmentation and the GVF-FCMs are compared with their counterparts segmented manually by the radiologists. Pearson's correlation coefficient (Pearson's *r*) and Paired Student's *t*-test are used to evaluate the consistency between computerized and manual segmentation. In the following discussion, *A*_*C*_ and *A*_*R*_ denote the lesion area calculated by computer and radiologists for a given lesion, respectively. *A*_*C*_∩*A*_*R*_ means an intersection set of the lesion areas returned from both methods, while *A*_*C*_ ∪ *A*_*R*_ means a union set. AOR_1_ and AOR_2_ are defined as two overlapping measures to compare the computerized segmentation with the radiologists' delineation [10, 13] as follows:

(12) $$\begin{array}{c} \text{AO}{\text{R}}_{\text{1}}=\frac{A_{C}\cap A_{R}}{A_{R}},\\ \text{AO}{\text{R}}_{\text{2}}=\frac{A_{C}\cap A_{R}}{A_{C}\cup A_{R}}. \end{array}$$

We calculate the AOR_1_ and AOR_2_ to evaluate the segmentation performance of the FCMs-based initial segmentation and the GVF-FCMs methods, respectively. Generally, a better segmentation attains when the AOR value approaches one.

### 2.6. Fisher Stepwise Discriminant Analysis Model

Discriminant analysis involves deriving a variate, which is a linear combination of the independent variables that would discriminate the best from a priori defined groups [30]. The method transforms the coordinates of the initial data to realize the least overlapping of the projections of data points in different groups for maximizing the diagnostic accuracy.

### 2.7. Statistical Analysis

The FSDA involves entering and removing features to get a statistically significant subset that predicts malignancy well, according to the discriminatory power of the subset adding to the group membership prediction [30]. Referring to [31], we set the value of the entering critical probability and the removal critical probability as *P* = 0.10 and *P* = 0.15, respectively. The FSDA is used to do the selection and classification of the features. In this study, a single database has been used for both training and testing, with the use of a “leave-one-out cross validation” method to avoid overfit. All the diagnostic performance details were calculated by the “leave-one-out cross validation” method.

The accuracy of a model in making predictions is evaluated regularly using a ROC analysis. An ROC curve is generated by combining the true positive fraction (sensitivity) and false positive fraction (1-specificity) with different setting decision thresholds. The area under an ROC curve (AUC) is taken to estimate the classification accuracy. Generally, a larger AUC stands for a better predictive performance.

## 3. Results and Discussion

While an accurate delineation of lesions on breast MRI is crucial for diagnosis and associated image-guided biopsy, a slice-by-slice manual delineation by radiologists is both time-consuming and subject to interobserver and intraobserver variations [32]. Our current study involves both computerized segmentation and characterization. This study is aimed at overcoming these problems.

### 3.1. Segmentation Performance

Table 1 summarizes the mean values and standard deviations of the areas from the lesion contours which were segmented by the FCMs-based, GVF-FCMs and the radiologists' manual delineation, respectively. The differences between the computerized method and radiologists' manual delineation are analyzed using the Pearson's correlation coefficient (Pearson's *r*) and Paired Student's *t*-test (Table 1). The original hypothesis is that there is no significant difference between the two groups of lesion areas segmented by different methods.

Pearson's *r* between the lesion areas segmented by the FCMs-based method and the radiologists' manual delineation was 0.891 while the paired *t*-test between the areas extracted by the two methods achieves a *P* value of 0.105. The result indicates that the areas worked out by the two methods are highly correlated without a significant difference at the averages. After refined by the GVF method, the *r* and *P* values were both increasing, which still showed highly correlation between the areas without a significant difference at averages (*P* > 0.05). These results indicate that both the two computerized methods have certain potentials to help radiologists in an accurate delineation, and the GVF-FCMs method showed the better performance among the two methods.

**Figure 3:**
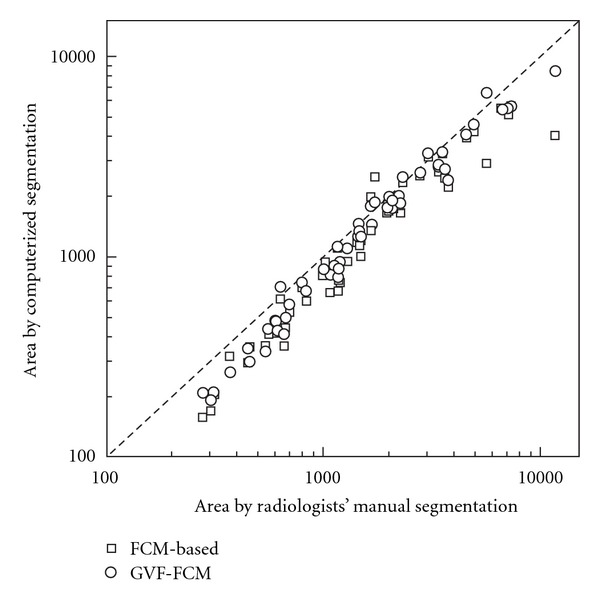
Scatter plot of the lesion areas segmented by computerized and radiologists' manual delineation. The diagonal line is represented the most perfect segmentation performance. *Square* is for areas segmented by FCMs-based initial method, *circle* is for areas extracted from GVF-FCMs method.

Figure 3 shows the log-log scatter plot of the areas measured using the computerized method versus radiologists' manual segmentation. The lesion area is the pixels numbers in the lesion region. We drew the log-log scatter plot because the range of lesion area is wide. Judged by the distribution of the data points in Figure 3, the computerized methods have somewhat underestimated the lesion area when compared with the radiologists' reference area, since the most of the data points are distributed below the reference diagonal line. The GVF-FCMs method has the smaller underestimated. One drawback of the FCMs implementation is that the method depends simply on the intensity information and does not include the pixels' spatial relationships. For a more complicated lesion enhancement, it is difficult for the FCMs-based method to locate the contour that approaches near to the realistic lesion contour. The GVF-FCMs method improves the initial segmentation when deforming to a balance of internal and external forces.

**Figure 4:**
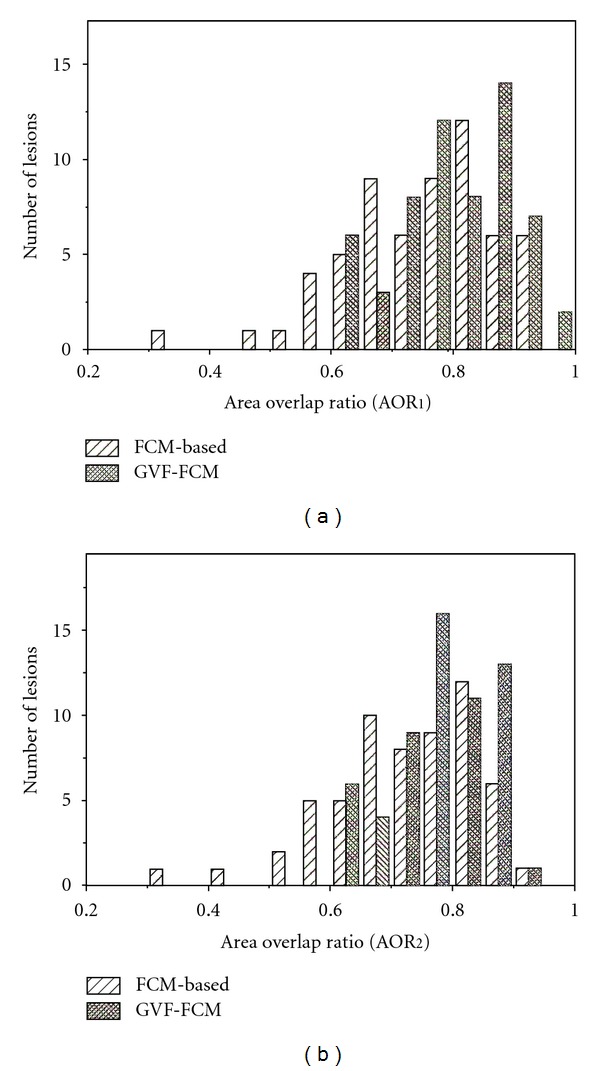
Histograms of the overlap measures on computerized methods: (a) AOR_1_; (b) AOR_2_. The closer the AOR value approximates to one, the better the segmentation performs. The GVF-FCMs method has the better performance among the two methods.

Figure 4 exhibits the histograms of the overlap measures on the computerized methods: the FCMs-based and the GVF-FCMs. It turns out that all lesions segmented by the GVF-FCMs method have the values of AOR_1_ and AOR_2_ over 0.6. The GVF-FCMs method has the better performance in overlap measures, too. From [10], 3D segmentation over the threshold value 0.4 indicates that this method has a successful segmentation of the lesion. The threshold should be stricter in 2D segmentation and is set to 0.6. At the overlap threshold, mass lesions were all segmented correctly after the refinements by GVF method. Two sets of overlap value were compared by using the Paired Student's *t*-test, and the *P* value between AOR_1_ was 0.064, while AOR_2_ was 0.005. AOR_2_ values were found to be statistically significant in average between the two computerized segmentation methods (*P* < 0.05).

### 3.2. Feature Selection and Performance of the Fisher Stepwise Discriminant Analysis Model

For the computerized characterization part, morphological and texture features are assessed to find out whether they can be used for classifying breast lesions, and whether the features from computerized segmentation method can have a better diagnostic performance in discriminating between benign and malignant lesions. Within the two training sets, features extracted by the two methods both had no statistically significant correlations between pairs of features.

#### 3.2.1. Features Extracted by GVF-FCMs Method

Among two computerized segmentation methods, the GVF-FCMs method achieves the better segmentation performance. Thus GVF-FCMs method is therefore adopted in the following analysis as a preferred method for the computerized characterization. When morphological features are taken into account alone, the classifier involves three features: spiculation, eccentricity, and solidity, with an AUC of 0.883. When using GLCM texture features, however, the classifier contains four features: entropy, difference average, difference variance and information measure of correlation 1, and the classifier could attain an AUC of 0.921. When combining all the morphological and texture features, five features were selected by the classifier with the improved AUC of 0.968. They were entropy, correlation, sum average, difference average and solidity. The diagnostic measure details are shown in Table 2.

#### 3.2.2. Features Extracted by Radiologists' Manual Delineation

The classifier selects only one morphological feature: spiculation with an AUC of 0.836. In view of the GLCM texture features, the classifier selects three features: entropy, difference average, and information measure of correlation 1 for ROC analysis with an AUC of 0.914. When combining the morphological and texture features, only the three aforementioned texture features were selected without any morphological feature. So the AUC was the same as only using texture features. The details of diagnostic performance are given in Table 2.

#### 3.2.3. Comparison of the Diagnostic Performance Based on Computerized and Manual Segmentation Methods

Different morphological features are selected when using different segmentation methods. Spiculation, eccentricity, and solidity are selected when GVF-FCMs segmentation method is applied, whereas only the spiculation is selected by means of radiologists' delineations. These features are both weighting the irregularity of the contour. Generally, a spiculated contour and irregular shape are attributed to a malignant lesion while smooth contour and circle-like shape are attributed to a benign one. The computerized segmentation method can improve the discriminatory power of morphological features, comparing with the results from radiologists' delineations.

When considering texture features, the features selected by the two segmentation methods are nearly the same. entropy, difference average, and information measure of correlation 1 are all selected by the two methods, but difference variance only selected by the computerized method. Entropy is related with the heterogeneity and complexity of lesion texture. The texture feature is presumably associated with a smooth margin, homogeneous, and lower enhancements of a benign lesion in comparison with an irregular margin, heterogeneous, and higher enhancements of a malignant lesion. The diagnostic performance is similar between the texture features from different segmentation methods.

By combining the morphological and texture features, none of morphological features is selected based on the radiologists' delineation while solidity is selected by the computerized segmentation method. This possibly could be due to the coarse polygon-like contour delineated by radiologists, and the morphological features only have moderate discriminatory powers. Since the GVF-FCMs method involves stretching and bending contour until the force balance, it can fit in with the real lesion contour well, and therefore the features from the GVF-FCMs method are more eligible for the classification of a breast lesion.

### 3.3. Comparison of the Areas under the ROC Curve

**Figure 5:**
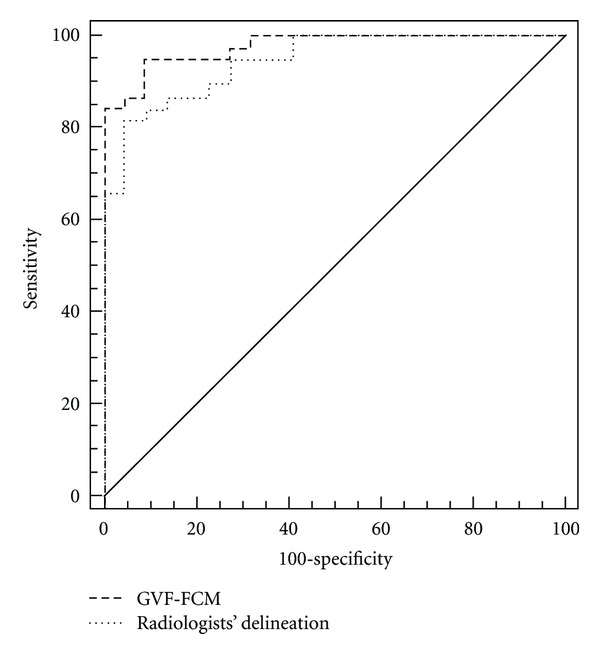
The ROC curves of classifer based on FSDA method by different features extracted by GVF-FCMs and radiologists' manual segmentation methods, respectively. The dotted line represented the ROC curve from radiologists' manual segmentation method with AUC of 0.914. The dash line denoted the ROC curve from computerized method (GVF-FCMs) with AUC of 0.968.

Figure 5 displays the ROC curves of the two discriminant functions. Applying the method by Delong et al. [33], no significant difference on the two AUCs is observed between the two classifiers (*P* = 0.231). The result yields two implications: firstly, the features extracted by the computerized segmentation method have the similar discriminant power with the situation when the contour is given by radiologists; secondly, the computerized characterization of a lesion probably provides a more efficient and objective method to quantify both the appearance (texture) and shape (morphology) features.

## 4. Conclusion

In this study, we have developed an approach based on FCMs clustering and the GVF snake model for mass-like lesion contour segmentation and computerized characterization on breast MRI. The segmentation performance measures show that the two step computerized segmentation method is an accurate method to automatically determine a suspicious lesion region and can help radiologists in their detection and delineation of breast MRI. At the computerized characterization part, Fisher stepwise discriminant analysis is used to select morphological and texture features and make classifications with the use of a “leave-one-out cross validation” method. The predictive performance based on the GVF-FCMs segmentation is better than the radiologists' manual method, but the difference is insignificant with the use of ROC curve analysis. The application of the breast MRI computerized segmentation and characterization package we developed may help radiologists to quantify the morphological and texture features and improve the objectivity and efficiency in interpreting breast MRI. In future, we intend to do further verification and assessment on a larger independent database.

## Figures and Tables

**Table 1:** Areas, statistical comparisons and area overlap measures of computerized delineation and radiologists' manual delineation.

Segmentation method	Area (mean ± SD pixels)	Pearson's correlation	*t*-test *P* value	AOR_1_ (mean ± SD)	AOR_2_ (mean ± SD)
FCM-based	1599.5 ± 1355.4	0.891	0.105	0.75 ± 0.13	0.72 ± 0.12
GVF-FCM	1815.3 ± 1722.2	0.976	0.437	0.81 ± 0.10	0.78 ± 0.08
Radiologists' manual	2114.9 ± 2093.8	—	—	—	—

**Table 2:** Diagnostic performance details of the segmentation by computerized and manual delineation methods.

Segmentation Method	Features	Accuracy (%)	Sensitivity (%)	Specificity (%)
GVF-FCM	Morphology (three selected)	83.3	84.2	81.2
	GLCM (four selected)	86.7	86.8	86.3
	Combing all features (five selected)	88.3	86.8	90.9

Radiologists' manual	Morphology (one selected)	75.0	73.7	77.3
	GLCM (three selected)	81.7	84.2	77.3
	Combing all features (three selected)	81.7	84.2	77.3

## Appendices

### A. Fuzzy c-Means Clustering Algorithm

The fuzzy c-means clustering is an unsupervised learning method in pattern recognition [23]. The algorithm is used to minimize the objective function *J* as follows:

(A.1) $$\begin{array}{c} J=\sum_{k=1}^{c}\;\sum_{i=1}^{N}u_{ki}^{b}{||x_{i}-c_{k}||}^{2}, \end{array}$$

with constraints

(A.2) ∑k=1cuki=1, 1≤i≤N,uki∈[0,1], 1≤i≤N,  1≤k≤c,0<∑i=1Nuki  <N, 1≤k≤c,

where *N* is the number of pixels in the region of interest (ROI); *c* is the number of clusters (the value is set to 2); *b* is a weighting exponent (A.2); **x**_*i*_ is the gray-level of a pixel in the ROI; **c**_*k*_ is the cluster center (start with random assignment); *u*_*ik*_ is the likelihood that the pixel **x**_*i*_ belongs to cluster *j*; the ||·|| denotes the Euclidean distance.

The objective function may minimize only if

(A.3) uki=1∑j=1c(||xi−ck||/||xi−cj||)2/(b−1), 1≤i≤N,  1≤k≤c,

(A.4) $$\begin{array}{c} c_{k}=\frac{\sum_{i=1}^{N}u_{ki}^{b}x_{i}}{\sum_{i=1}^{N}u_{ki}^{b}}. \end{array}$$

The values of *u*_*ki*_ and **c**_*k*_ will iteratively update with the (A.3) and (A.4). The iteration will stop when the stop criterion is reached (|*J*_*n*+1_ − *J*_*n*_| ≤ 0.0005) or the max iteration is reached (100).

### B. Gray-Level Cooccurrence Matrix (GLCM) and Features Extracted from GLCM

Spatial gray-level cooccurrence matrix estimates image properties related to the second-order statistics [15]. Each element (*i*, *j*) in GLCM specifies the number of times that the pixel with gray-level value *i* occurred adjacent to a pixel with value *j* at a given offset (Δ*x*, Δ*y*). Mathematically, GLCM element over an image *S* is given as

(B.1) $$\begin{array}{c} p\left(i,j\right)=\frac{\#\left\{\left[\left(x,y\right),\left(x+\Delta x,y+\Delta y\right)\right]\in S\;|\;f\left(x,y\right)=i,f\left(x+\Delta x,y+\Delta y\right)=j\right\}\;}{\#S}, \end{array}$$

where # represented the number of specific pixel-pair. If *N* is the number of distinct gray levels of an image, we denote that

(B.2) $$\begin{array}{c} p_{x}\left(i\right)=\sum_{j=1}^{N}p\left(i,j\right),\;i=1,2,\ldots ,N,\\ p_{y}\left(j\right)=\sum_{i=1}^{N}p\left(i,j\right),\;j=1,2,\ldots ,N,\\ p_{x+y}\left(k\right)=\sum_{i=1}^{N}\;\sum_{j=1}^{N}p\left(i,j\right),\;k=i+j=2,3,\ldots ,2N,\\ p_{x-y}\left(k\right)=\sum_{i=1}^{N}\;\sum_{j=1}^{N}p\left(i,j\right),\;k=\left|i-j\right|=0,1,\ldots ,N-1. \end{array}$$

Then the thirteen texture features are calculated as follows.

*f*_1_: Angular second moment
  (B.3) $$\begin{array}{c} f_{1}=\sum_{i}\sum_{j}p{\left(i,j\right)}^{2}. \end{array}$$
  *f*_2_: Contrast
  (B.4) $$\begin{array}{c} f_{2}=\sum_{k=0}^{N-1}k^{2}p_{x-y}\left(k\right). \end{array}$$
  *f*_3_: Correlation
  (B.5) $$\begin{array}{c} f_{3}=\frac{1}{\sigma_{x}\sigma_{y}}\sum_{i=1}^{N}\;\sum_{j=1}^{N}\left(ij\right)p\left(i,j\right)-\mu_{x}\mu_{y}, \end{array}$$
  where *μ*_*x*_ and *σ*_*x*_ are the mean and standard deviations of *p*_*x*_, respectively; *μ*_*y*_ and *σ*_*y*_ are the mean and standard deviations of *p*_*y*_, respectively.
*f*_4_: Inverse difference moment
  (B.6) $$\begin{array}{c} f_{4}=\sum_{i}\sum_{j}\frac{1}{1+{\left(i-j\right)}^{2}}p\left(i,j\right). \end{array}$$
  *f*_5_: Sum average
  (B.7) $$\begin{array}{c} f_{5}=\sum_{k=2}^{2N}kp_{x+y}\left(k\right). \end{array}$$
  *f*_6_: Sum variance
  (B.8) $$\begin{array}{c} f_{6}=\sum_{k=2}^{2N}{\left(k-f_{5}\right)}^{2}p_{x+y}\left(k\right). \end{array}$$
  *f*_7_: Sum entropy
  (B.9) $$\begin{array}{c} f_{7}=-\sum_{k=2}^{2N}p_{x+y}\left(k\right)\log\left(p_{x+y}\left(k\right)\right). \end{array}$$
  *f*_8_: Entropy
  (B.10) $$\begin{array}{c} f_{8}=-\sum_{i}\sum_{j}p\left(i,j\right)\log\left(p\left(i,j\right)\right).\; \end{array}$$
  *f*_9_: Difference average
  (B.11) $$\begin{array}{c} f_{9}=\sum_{i=1}^{N}{\left(i-\mu \right)}^{2}p_{x}\left(i\right), \end{array}$$
  where *μ* is the average of *p*(*i*, *j*).
*f*_10_: Difference variance
  (B.12) $$\begin{array}{c} f_{10}=\sum_{k=0}^{N-1}\left(k-d\right)p_{x-y}\left(k\right), \end{array}$$
  where *d* is the mean of *p*_*x*−*y*_.
*f*_11_: Difference entropy
  (B.13) $$\begin{array}{c} f_{11}=-\sum_{k=0}^{N-1}p_{x-y}\left(k\right)\log\left(p_{x-y}\left(k\right)\right). \end{array}$$
  *f*_12_: Information measure of correlation 1
  (B.14) $$\begin{array}{c} f_{12}=\frac{f_{9}+\sum_{i}\sum_{j}p\left(i,j\right)\log\left(p_{x}\left(i\right)p_{y}\left(j\right)\right)\;}{\underset{}{\max}\left(-\sum_{j}p_{x}\left(i\right)\log\left(p_{x}\left(i\right)\right),-\sum_{j}p_{y}\left(i\right)\log\left(p_{y}\left(i\right)\right)\right)}. \end{array}$$
  *f*_13_: Information measure of correlation 2
  (B.15) $$\begin{array}{c} f_{13}=\sqrt{1-\exp\left(-2\left(E-f_{8}\right)\right)}, \end{array}$$
  where *E* = −∑_*i*_∑_*j*_ *p*_*x*_(*i*)*p*_*y*_(*j*)log⁡⁡(*p*_*x*_(*i*)*p*_*y*_(*j*)).
